# Trust in COVID-19 policy among public health professionals in Israel during the first wave of the pandemic: a cross-sectional study

**DOI:** 10.1186/s13584-022-00529-6

**Published:** 2022-04-11

**Authors:** Tamar Zohar, Maya Negev, Maia Sirkin, Hagai Levine

**Affiliations:** 1 School of Public Health, University of Haifa, Haifa, Israel; 2 School of Public Health, Hebrew University-Hadassah, Jerusalem, Israel

**Keywords:** COVID-19, Decision making, Health policy, Pandemic, Public health, Trust

## Abstract

**Background:**

The COVID-19 pandemic has highlighted the important role of professionals in designing and communicating effective policies. The purpose of this study was to evaluate the level of trust in the COVID-19 national public health policy among public health professionals in Israel and its correlates during the first wave of the pandemic.

**Methods:**

A purposive sampling of public health professionals in Israel, through professional and academic public health networks (N = 112). The survey was distributed online during May 2020. Level of trust was measured by the mean of 18 related statements using a 5-point Likert scale, where 1 means not at all and 5 means to a very high extent, and grouped as low and high trust by median (2.75).

**Results:**

A moderate level of trust in policy was found among professionals (Mean: 2.84, 95% Cl: [2.70, 2.98]). The level of trust among public health physicians was somewhat lower than among researchers and other health professionals (Mean: 2.66 vs. 2.81 and 2.96, respectively, *p* = 0.286), with a higher proportion expressing low trust (70% vs. 51% and 38%, respectively, *p* < 0.05). Participants with a low compared to high level of trust in policy were less supportive of the use of Israel Security Agency tools for contact tracing (Mean = 2.21 vs. 3.17, *p* < 0.01), and reported lower levels of trust in the Ministry of Health (Mean = 2.52 vs. 3.91, *p* < 0.01). A strong positive correlation was found between the level of trust in policy and the level of trust in the Ministry of Health (rs = 0.782, *p* < 0.01). Most professionals (77%) rated their involvement in decision making as low or not at all, and they reported a lower level of trust in policy than those with high involvement (Mean = 2.76 vs. 3.12, *p* < 0.05). Regarding trust in the ability of agencies to deal with the COVID-19 crisis, respondents reported high levels of trust in the Association of Public Health Physicians (80%) and in hospitals (79%), but very low levels of trust in the Minister of Health (5%).

**Conclusions:**

This study shows that Israeli public health professionals exhibited moderate levels of trust in COVID-19 national public health policy and varied levels of trust in government agencies during the first wave of COVID-19. The level of trust in policy was lower among most of the participants who were not involved in decision making. The level of trust found is worrisome and should be monitored, because it may harm cooperation, professional response, and public trust. Professionals’ trust in policy-making during early stages of emergencies is important, and preemptive measures should be considered, such as involving professionals in the decision-making process, maintaining transparency of the process, and basing policy on scientific and epidemiological evidence.

**Supplementary Information:**

The online version contains supplementary material available at 10.1186/s13584-022-00529-6.

## Background

The coronavirus (COVID-19) pandemic is one of the most severe public health crises to occur in recent history. On January 30th 2020, the COVID-19 outbreak was declared a public health emergency of international concern by the World Health Organization (WHO) [1]. By March 2020, the virus had spread to almost all countries around the world, and on March 11^th^ 2020, the COVID-19 outbreak was declared a pandemic [2]. With uncertainty surrounding the source and spread of the virus, many countries and organizations initially relied solely on WHO guidelines, rather than constructing their own [3]. As the pandemic persisted, countries began to create guidelines that matched the needs of their populations with the assistance of public health professionals (hereafter, ‘professionals’).

Israel is a democratic country in the Middle East [4], with 9.3 million people [5], and a centralized public health system administered by the Ministry of Health [6, 7]. Israel was one of the first countries to respond to the crisis by putting guidelines and restrictions in place in response to the virus. These guidelines included lockdowns, social distancing precautions such as closing schools and forbidding public gatherings, and new technological methods for contact tracing [8] which increased government monitoring of citizens. The contact tracing was conducted by the surveillance system of the Israeli Security Agency (ISA), which has expertise in national security and monitoring terrorism. Following the adoption of emergency regulations, the ISA was activated to assist the national effort to reduce the spread of COVID-19 via large-scale surveillance of civilians for the purposes of COVID-19 contact tracing [9, 10].

The COVID-19 outbreak has highlighted the importance of creating and communicating effective public health policies. A major determining factor in the success of a public health intervention is trust, both from the public and among decision makers. Studies on trust in government and on previous epidemics have shown that trust in the government is an important determinant of a population’s compliance with public health policies and guidelines [11, 12]. The level of public trust influences the response of the public to the threat of an infectious disease and its acceptance of health information, ultimately determining the success of a public health intervention [13]. If decision-making processes do not seem justified or transparent, public trust can be threatened, putting an emerging public health intervention in jeopardy [14]. It has been suggested that one way to increase government trust is to use credible information intermediaries, such as experts in the field, to increase the credibility of information and subsequently increase public trust and motivation to comply with health policies [15]. With this in mind, professionals have an important and unique role in mediating public trust toward the government and among public health leaders during public health emergencies. For this reason, it is crucial to evaluate and promote trust in national policies among professionals, in order to encourage public trust and create successful public health interventions.

In emergency events, public health officials are required to make rapid decisions to maintain public health [16, 17]. In the case of an emerging infectious disease such as COVID-19, these policies need to be created and evaluated under strict time constraints, and with limited information. There are many tools and strategies used by public health officials to make quick and effective decisions. Professionals have expertise in these decision-making strategies, specifically in evidence-based research, which is commonly used in response to an infectious disease outbreak [18, 19]. Since professionals are trained in evidence-based research, they play a key role in facilitating evidence-based decisions in the development of health policy. Additionally, an important aspect of evidence-based research and decision making includes access to credible sources. Understanding which sources are used and trusted by professionals facilitates the evaluation of credibility of organizations involved in making policy and increases the transparency of the decision-making process.

A public health professional can be defined as someone who studied or works in the field of public health, which may include a range of related health fields such as medicine, nursing, research, nutrition etc. The demographics, primary occupations, and seniority levels among professionals can vary greatly and may contribute to differences in practice and opinions. A report on the views of public officials from the Chinese Centers for Disease Control (CDC) found that senior Chinese CDC staff displayed less confidence in their surveillance systems for infectious disease than lower level staff [20]. This suggests that the professional status of professionals may affect their reported perceptions of interventions. Similarly, a study reviewing risk perception among Japanese healthcare workers during the SARS outbreak in 2002–2004 highlighted differences in perception between physicians and nurses; nurses displayed a higher level of preventative measures, while physicians demonstrated a greater acceptance of risk. This again emphasizes the variation in perception among different professionals regarding public health practices [21].

Although there have been many studies of public opinion on public health guidelines and policies, little research has been done on the perceptions and opinions of professionals in response to country-wide public health interventions or epidemics. Moreover, the level of trust among professionals was not known at the beginning of the COVID-19 pandemic. Professionals in this field often play an important role in both public health decision making and influencing public trust, since the success of a public health response during a pandemic relates to public trust in experts, and on the public believing that these scientists are involved in decision-making processes [22, 23]. In recent years, the level of public confidence in politicians and decision-makers has declined worldwide [22] and within Israeli society [24]. The mistrust demonstrated by professionals towards policies may have exacerbated public mistrust of these policies. Therefore, it is important to better understand whether the public’s low trust in decision makers also appears among professionals, by examining professionals’ level of trust in decision makers, government agencies, and policies.

Moreover, examining professionals’ trust in COVID-19-related health policy has the potential to provide valuable lessons for improving management of future large-scale emergencies such as pandemics, particularly in the early stages when an immediate response is needed. Accordingly, the goal of the current study was to evaluate Israeli professionals’ level of trust in COVID-19 national public health policy (hereafter, ‘policy’) and its correlations, during the first wave of the pandemic. Specifically, to assess the level of trust in policy and its correlation to personal compliance with COVID-19 guidelines, perceptions regarding the use of ISA tools for contact tracing and monitoring morbidity, socio-demographic factors (position, seniority, age, gender, and religion) of participants, involvement in decision-making processes, level of trust in the various agencies dealing with the COVID-19 crisis and level of credibility of sources of information (study framework in Additional file 1).

We hypothesized that the level of trust will be correlated with personal compliance with COVID-19 guidelines, perceptions regarding the use of ISA tools, socio-demographic factors, involvement in decision making, the level of trust towards the various agencies and credibility of sources of information.

## Methods

We performed an online cross-sectional study. A survey instrument was developed to evaluate perceptions and attitudes towards policy among professionals in Israel during the first wave of the COVID-19 pandemic. A pilot study was conducted to ensure readability and validity by sending the draft survey instrument to several people who provided feedback on the quality and clarity of the questions; it was then corrected according to these comments before being distributed. The survey was distributed online during the first 2 weeks of May 2020 using a Qualtrics XM online survey to obtain rapid responses from professionals in Israel during the first wave of the COVID-19 pandemic. The use of an online survey made it compliant with the social distancing restrictions that were in force during this time period. The survey was in Hebrew and all answers were recorded anonymously in the Qualtrics system. In order to reach professionals, purposive sampling [25–27] was conducted among professionals in Israel. To obtain a broad sample of professionals, an effort was made to contact all relevant agencies involved in public health and the questionnaire was distributed through relevant lists and social networks of the Ministry of Health, Schools of Public Health, and the Israel Association of Public Health Physicians (which is the official scientific association of public health professionals in Israel). A general request was made online through these communication networks for voluntary participation in the study by answering a directed questionnaire of approximately 15 min in length. The survey was approved by the Ethics Committee of the Faculty of Social Welfare and Health Sciences, University of Haifa. Online informed consent was obtained from all participants, participation was voluntary, and all data and information were kept anonymous.

A total of 227 participants entered the survey platform. However, 66 (29%) participants entered only the first page of the consent document in the survey and 49 (21%) surveys were not fully completed. Those surveys were omitted from the sample, leaving a total of 112 eligible participants, with each respondent only able to participate in the survey once. The questions about credibility of sources of information were at the beginning of the survey and were also answered by the 49 participants who didn't complete the survey. There were no significant differences in credibility of sources of information between those who completed the survey and those who did not.

The structured survey (Additional file 2) included questions regarding: (1) Credibility of the source of information and frequency of use; (2) Self-involvement in decision making during the COVID-19 outbreak; (3) Perceptions of the decision-making process during the COVID-19 outbreak; (4) Level of compliance with the COVID-19 guidelines; (5) Perceptions of credibility of various agencies dealing with COVID-19; (6) Evaluation of the quality of measures adopted to maintain public health; (7) Perceptions regarding the use of ISA tools for contact tracing; and (8) Socio-demographic information, such as: age, gender, profession, level of religiosity etc. Most of the questions were on a 5-point Likert scale from 1 (not at all) to 5 (high agreement).

Level of trust in policy among professionals was calculated as a numerical value for an average score from the 5-point Likert scale for 18 related statements (Cronbach α = 0.95) regarding perceptions of the decision-making process, evaluation of the quality of guidelines adopted to maintain and protect public health, and transparency during the decision-making process, such as: "Decisions were made based on data from local and global information", "The measures requested by the Ministry of Health are important for reducing or preventing the spread of COVID-19", "The official guidelines were given clearly and were accompanied by an appropriate information system", "The official guidelines were based on professional logic" and "The decision-making process was conducted transparently". The level of trust in policy was divided into two categories: participants with higher than median (2.75) level of trust were merged into a high level of trust in policy category and participants with lower than and equal to median levels of trust were merged into a low level of trust in policy category.

Personal compliance with national guidelines was a numerical value for the average of four related questions covering the level of compliance with guidelines published regarding COVID-19, maintaining a distance of 2 m between people, and wearing a mask in public spaces and at work (Cronbach α = 0.81).

Perceptions regarding the use of ISA tools for contact tracing were a numerical value that calculated as an average score on the 5-point Likert scale from 1 (not at all) to 5 (very high) for four related statements (Cronbach α = 0.79), such as: "There was justification for using Security Agency tools to locate patients in order to reduce/prevent infection by COVID-19" and "There was justification for using Security Agency tools to locate patients even at the cost of violating the rights and privacy of the citizen".

Involvement in decision making was categorial value that calculated from responses on a 5-point Likert scale to one main statement: "To what extent are you involved in decision-making processes?". Level of involvement in decision-making was calculated by merging scores 1 and 2 (representing not at all and low level of involvement) from the question into a low level of involvement in decision-making category and by merging scores 3–5 (representing medium, high, and very high level of involvement) into a high level of involvement category.

Involvement in decision making was categorial value that calculated from responses on a 5-point Likert scale to one main statement: "To what extent do you participate in the discussions in which decisions are made?" Level of involved in discussions was calculated by merging scores 1 and 2 (representing not at all and low level of involvement) from the question into a low level of involvement in discussions category and merging 3–5 (representing medium, high, and very high level of involvement) into a high level of involvement in discussions category.

The level of trust in the various agencies dealing with the COVID-19 crisis for each agency is presented as a frequency of the total number of answers in each category on a 5-point Likert scale from 1 (not at all) to 5 (very high) in response to the statement: "Please describe the level of trust you have in the following agencies’ ability to deal with the COVID-19 pandemic".

Level of perceived credibility of sources of information was calculated as an ordinal value for each source of information on a 5-point Likert scale from 1 (not at all) to 5 (very high).

Credibility of sources of information is presented as the frequency of high and very high scores on a 5-point Likert scale for one statement, "To what extent do you trust the reliability of information about the COVID-19 pandemic, from any of the following sources?" and frequency of use is presented as the frequency of daily use or several times a day scores on a 5-point Likert scale for one statement, "How often are you updated on information about the COVID-19 pandemic, from any of the following sources?".

### Statistical analyses

A Mann–Whitney U test was performed to assess the differences between the participants that completed the survey compere and those who did not, regarding the questions about credibility of sources of information.

One-way ANOVA was used to compare mean level of trust in policy between public health physicians, researchers and other health professionals. A chi-square test of independence was used to assess the association between low and high levels of trust in policy and public health professionals (public health physicians, researchers and other health professionals), professional seniority in public health (below and above 10 years), gender (male and female), religion (Jewish and other), level of religiosity (secular and other), and level of involvement in decision-making processes (low and high level of involvement). For significant association between low and high levels of trust in policy and public health profession, we also present the percentage of low and high level of trust in policy for every profession category. A Mann–Whitney U test was used to compare differences between low and high levels of trust in policy and trust in the Ministry of Health, Minister of Health and Prime Minister. An independent-samples t-test was used to compare professionals with a low level of trust in policy to those with a high level of trust in policy, with respect to mean age, perceptions regarding the use of ISA tools and personal compliance.

The Pearson correlation coefficient was used to assess two different relationships: (1) the relationship between personal compliance with guidelines and the level of trust in policy among professionals: and (2) the perceptions regarding the use of ISA tools and the level of trust in policy among professionals.

Univariate tests were performed to examine the relationship between sociodemographic characteristics and level of trust in policy. The Pearson correlation coefficient was used to assess the relationship between age and level of trust in policy. An independent-samples t-test was used to compare the level of trust in policy between categories within the following variables: seniority (below and above 10 years), gender (male and female), level of religiosity (secular and other). A multiple linear regression was performed to predict the level of trust in policy among professionals based on profession, seniority, age, gender, and level of religiosity, where gender was coded as 0 = male, 1 = female, level of religiosity was coded as 0 = secular, 1 = religious, professional seniority was coded as 0 = below 10 years, 1 = above 10 years, profession was coded as 0 = public health physicians, 1 = other, and age was measured in years.

An independent-samples t-test was used to compare the difference in the level of trust in policy between professionals with a low level of involvement and those with a high level of involvement. Spearman's rho correlation coefficient was used to assess the relationships between two questions regarding the level of involvement in decision making and in discussions.

Spearman's rho correlation coefficient was used to assess three different relationships: (1) the relationship between the level of trust in the various agencies dealing with the COVID-19 crisis and the level of trust in policy among professionals; (2) the relationship between the perceived level of credibility of sources of information and the level of trust in policy among professionals; and (3) the relationship between the credibility of sources of information regarding COVID-19 and their frequency of use.

## Results

### Participants

A total of 112 participants completed 95–100% of the online survey; 33 (30%) were men and 75 (67%) were women. Ninety-four (84%) participants provided information about their age, which ranged from 29 to 82 years, with a mean of 48 years. Currently, there are approximately 163 public health physicians in Israel; 27 of them (17%) completed the survey. Twenty-seven (24%) participants were public health physicians (item “a” in question J.2, see Additional file 2), 42 (38%) were other health professionals (other than public health physicians) (d, e, i), 35 (31%) were researchers (b, c, f, g, h) and 8 (7%) were missing (Table 1).

**Table 1:** Socio-demographic characteristics of the participants

Variable*	Value	Distribution
Profession in public health	Public health physician	24.1%
	Other health professional	37.5%
	Researcher	31.3%
Highest degree in public health	PhD	20.5%
	MPH	39.3%
	MSc	12.5%
	MHA	6.3%
	Student of public health	11.6%
Professional seniority in public health	1–4 years	15.2%
	5–10 years	22.3%
	11–15 years	15.2%
	Over 16 years	46.4%
Gender	Male	29.5%
	Female	67.0%
Age	21–40	19.6%
	41–50	30.4%
	51–60	22.3%
	61–70	11.6%
	Minimum	29
	Maximum	82
	Mean	48
	SD	11.6
Religion	Jewish	89.0
	Muslim	4.6%
	Christian	5.5%
	Druze	0.9%
Level of religiosity	Secular	74.1%
	Traditional	8.9%
	Religious	12.5%
	Ultra-Orthodox	0.9%

*N = 112. Missing data: profession in public health—7.1%, highest degree in public health—9.8%, professional seniority in public health—0.9%, gender—3.5%, age—16.1%, level of religiosity—3.6%

### Level of trust

Level of trust in policy among professionals was found to be moderate (Mean = 2.84, SD = 0.76, Median = 2.76). The mean level of trust in policy among public health physicians (Mean = 2.66, SD = 0.69) was somewhat lower than among researchers (Mean = 2.81, SD = 0.78) and other health professionals (Mean = 2.96, SD = 0.78), but the differences among the groups were not significant (*p* = 0.286).There were no significant differences in the socio-demographic characteristics (professional seniority in public health, gender, religion and level of religiosity) of the participants with low vs. high levels of trust in policy, besides the difference by profession (Table 2). Notably, a higher proportion of public health physicians had low trust in policy compared to researchers and other health professionals (70% vs. 51% and 38%, respectively, *p* < 0.05). Participants with low trust in policy were less supportive of the use of ISA tools than those with high trust in policy (Mean = 2.21 vs. 3.17, *p* < 0.001). Similarly, participants with low trust in policy reported lower levels of trust in the Ministry of Health, the Minister of Health and the Prime Minister, than those with high trust in policy (Mean = 2.52 vs. 3.91, 1.23 vs. 1.89, and 1.59 vs. 2.64, respectively; *p* < 0.001 for all). There were no significant associations of age and other socio-demographic characteristics with the level of involvement in decision-making processes.

**Table 2:** Socio-demographic characteristics of the participants with low and high levels of trust in COVID-19 policy (by percentage) and comparison between the groups

Variable	Value	Trust in COVID-19 policy*			
		Low level (n = 56) [%]	High level (n = 56) [%]		*p*-value
Public health profession^a^					< 0.05
	Public health physician	35.8	15.7		
	Researcher	34.0	33.3		
	Other health professional	30.2	51.0		
Professional seniority in public health^a^					0.751
	1–4 years	12.7	17.9		
	5–10 years	23.6	21.4		
	11–15 years	18.2	12.5		
	Over 16 years	45.5	48.2		
Gender^a^					0.531
	Male	33.3	27.8		
	Female	66.7	72.2		
Religion^a^					0.741
	Jewish	89.1	87.0		
	Muslim	7.3	1.8		
	Christian	1.8	9.3		
Level of religiosity^a^					0.429
	Secular	80.0	73.6		
	Traditional	9.1	9.4		
	Religious	10.9	15.1		
	Ultra-Orthodox	0.0	1.9		
Involvement in decision-making processes^a^					0.654
	Low	78.6	75.0		
	High	21.4	25.0		

	Mean	SD	Mean	SD	**
Trust in Ministry of Health^b^	2.52	0.786	3.91	0.769	< 0.001
Trust in Minister of Health^b^	1.23	0.504	1.89	0.985	< 0.001
Trust in Prime Minister^b^	1.59	0.848	2.64	1.23	< 0.001
Age^c^	48.7	12.8	50.2	10.1	0.530
Use of Israeli Security Agency tools^c^	2.21	0.695	3.17	0.638	< 0.001
Personal compliance^c^	4.05	0.883	4.42	0.541	< 0.01

^*^Percentage of participants with low and high levels of trust in COVID-19 policy by socio-demographic characteristics

^**^Mean and standard deviation for levels of trust in the various agencies, age, use of Israeli Security Agency tools and personal compliance, between participants with low compared to high levels of trust in COVID-19 policy

^a^Pearson’s chi-squared test

^b^Mann–Whitney U test

^c^Independent samples t-test

### Personal compliance with guidelines

Personal compliance with national guidelines was relatively high (Mean = 4.2, SD = 0.75). A low positive correlation was obtained for the relationship between personal compliance and the level of trust in policy among professionals (R = 0.296, *p* < 0.01).

### Perceptions regarding the use of Israeli Security Agency tools

A strong positive correlation was obtained for the relationship between perceptions regarding the use of ISA tools for contact tracing and the level of trust in policy among professionals (R = 0.601, *p* < 0.001).

### Level of trust in COVID-19 policy by socio-demographic factors

The level of trust in policy among professionals in Israel was examined with respect to socio-demographic factors. The univariate tests that were performed to examine the relationship between sociodemographic characteristics and level of trust in policy found no significant difference for any of the variables. Similarly, age was not significantly correlated with the level of trust in policy. A multiple linear regression was calculated to predict the level of trust in policy among professionals based on profession, seniority, age, gender, and level of religiosity. The regression equation was not significant (F_5,81_ = 0.910, *p* = 0.479; R^2^ = 0.053) overall or for any of the variables.

### Involvement in decision-making processes

Most professionals (77%) rated their involvement in decision making as low. There was a lower level of trust among those with lower involvement compared to those with high involvement (M = 2.76, SD = 0.73 vs. M = 3.12, SD = 0.81, *p* < 0.05). Most professionals (76%) rated their involvement in discussions as low, and the responses to the two questions regarding involvement in decision making and in discussions were highly correlated (rs = 0.771, *p* < 0.001). There was a marginally lower level of trust among those with lower involvement in discussions compared to those with high involvement in discussions (M = 2.77, SD = 0.71 vs. M = 3.08, SD = 0.87, *p* = 0.064).

### Level of trust in the various agencies dealing with the COVID-19 crisis

**Fig. 1:**
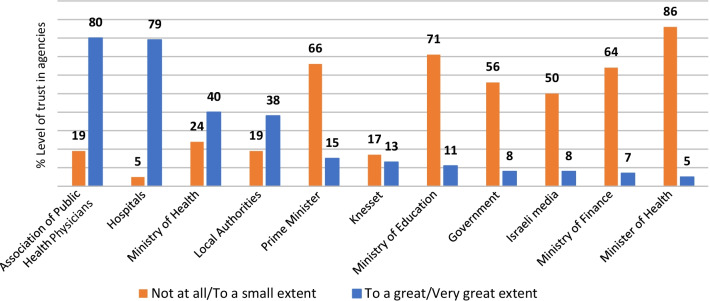
Proportions (%) of high (4–5 on a 5-point Likert scale) and low (1–2) levels of trust in agencies during the first outbreak of COVID-19

There were marked differences in participants’ evaluation of their level of trust in the ability of different agencies to deal with the COVID-19 crisis during the first wave of COVID-19 (Fig. 1). A very high percentage reported a high level of trust in the Association of Public Health Physicians (80%) and in hospitals (79%), compared to only 5% who reported a high level of trust in the Minister of Health, 8% in the government and 15% in the Prime Minster. Moreover, less than half (40%) reported a high level of trust in the Ministry of Health.

### Association between level of trust in agencies and level of trust in COVID-19 policy

**Fig. 2:**
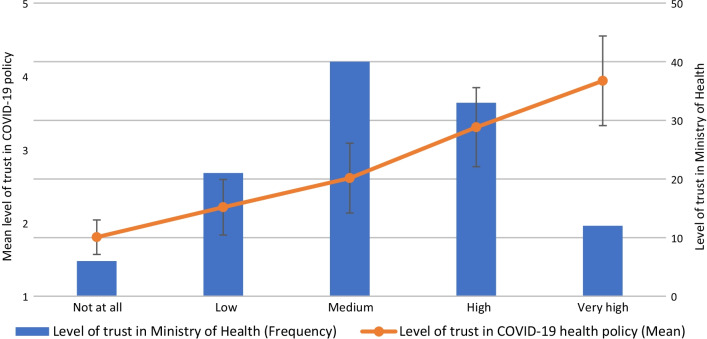
Association between level of trust in Ministry of Health and level of trust in COVID-19 policy

**Fig. 3:**
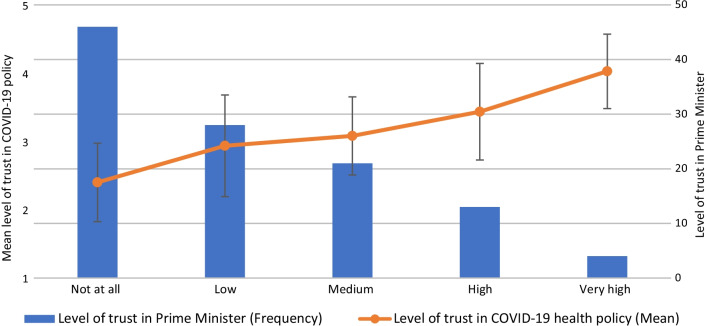
Association between level of trust in Prime Minister and level of trust in COVID-19 policy

**Fig. 4:**
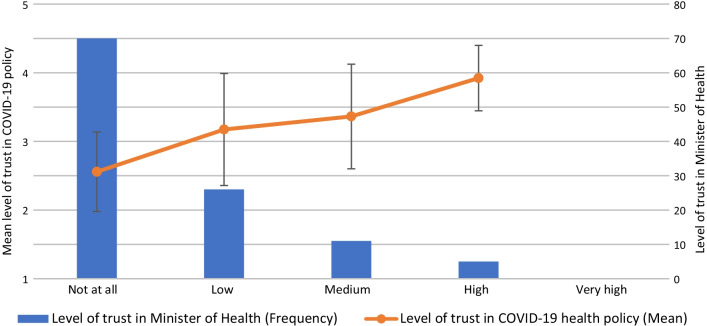
Association between level of trust in Minister of Health and level of trust in COVID-19 policy

The mean level of trust in policy is presented for each category on a 5-point Likert scale (Figs. 2, 3, 4). The results of the Spearman’s rho test between trust in policy and trust in various agencies’ ability to deal with the COVID-19 crisis (Table 3) indicate a significant positive relationship for the following agencies: Ministry of Health (r_s_ = 0.782, *p* < 0.01; Fig. 2), Prime Minister (r_s_ = 0.558, *p* < 0.01; Fig. 3), Minister of Health (r_s_ = 0.483, *p* < 0.01; Fig. 4), Government (r_s_ = 0.454, *p* < 0.01), Knesset (Israeli parliament) (r_s_ = 0.337, *p* < 0.01), Ministry of Education (r_s_ = 0.337, *p* < 0.01), Ministry of Finance (r_s_ = 0.303, *p* < 0.01) and Hospitals (r_s_ = 0.252, *p* < 0.01). No significant relationships were found between professionals’ level of trust in policy and their level of trust in other agencies, such as the Association of Public Health Physicians, the Israeli media and local authorities.

**Table 3:** Correlation between level of trust in various agencies and level of trust in COVID-19 policy

Authority*	Correlation coefficient	Significance (2-tailed)
Ministry of Health	0.782	< 0.001
Prime Minister	0.558	< 0.001
Minister of Health	0.483	< 0.001
Government	0.454	< 0.001
Knesset	0.337	< 0.001
Minister of Education	0.337	< 0.001
Ministry of Finance	0.303	< 0.001
Hospitals	0.252	< 0.001
Association of Public Health Physicians	0.134	0.158
The Israeli media	0.097	0.308
Local authorities	− 0.050	0.602

^*^N = 112

### Level of perceived credibility of sources of information and trust in policy

Spearman's rho correlation coefficient was used to assess the relationship between the level of credibility of sources of information and the level of trust in policy among professionals. A significant positive relationship was found between the level of trust in policy and the level of credibility of the following sources of information: Ministry of Health (r_s_ = 0.713, *p* < 0.01), Websites of government ministries in Israel other than the Ministry of Health (r_s_ = 0.391, *p* < 0.01), foreign government websites (r_s_ = 0.209, *p* < 0.05), Israeli media (r_s_ = 0.192, *p* < 0.05) and WHO (r_s_ = 0.189, *p* < 0.05). It was found that when the level of perceived credibility of these sources of information is higher, the level of trust in policy increases. No significant relationships were found between the level of trust in policy among professionals and other sources of information, such as: WhatsApp groups, the WhatsApp group "Public Health", academic journals, groups online or on social media.

### Sources of information: credibility and usage

**Fig. 5:**
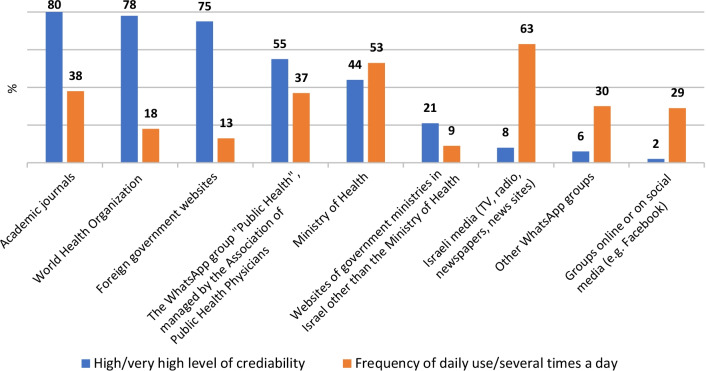
Relative credibility of sources of information and frequency of use

Descriptive statistics for credibility of sources of information and frequency of use are presented in Fig. 5. Although most professionals reported high or very high levels of credibility for academic journals (80%), WHO (78%), and foreign government websites (75%), only 38% reported a high frequency of use of academic journals, 18% of WHO, and 13% of foreign government websites. This is in contrast to Israeli media, for which only 8% reported high or very high levels of credibility, but 63% reported a high frequency of use.

The results of the Spearman's rho test between credibility and usage were significant for WhatsApp groups (r_s_ = 0.549, *p* < 0.05), the WhatsApp group "Public Health", managed by the Association of Public Health Physicians (r_s_ = 0.362, *p* < 0.01), foreign government websites (r_s_ = 0.347, *p* < 0.01), the Ministry of Health (r_s_ = 0.307, *p* < 0.01), the Israeli media (TV, radio, newspapers, news sites) (r_s_ = 0.289, *p* < 0.01), WHO (r_s_ = 0.271, *p* < 0.01), websites of government ministries in Israel other than the Ministry of Health (r_s_ = 0.263, *p* < 0.01), and groups online or on social media (r_s_ = 0.219, *p* < 0.05). There was no significant correlation between credibility and usage for academic journals (r_s_ = 0.095, *p* = 0.0318).

## Discussion

This study found a moderate level of trust in policy among professionals. The proportion of professionals with a low level of trust in policy was especially high among public health physicians, while a higher level of trust in policy among professionals was found for those with a higher level of involvement in decision-making processes. Notwithstanding these differences, reported personal compliance with COVID-19 guidelines was relatively high among all responders. A strong positive correlation was found between perceptions regarding the use of ISA tools and the level of trust in policy. Similarly, strong positive associations were found between the level of trust in policy and the level of trust in the Ministry of Health and the Prime Minister. In contrast, no correlation was found between the level of trust in policy and professionals’ socio-demographic factors.

These results can contribute to further improving the management of public health policy and credibility of national agencies, from the perspective of professionals, especially during future large-scale emergencies such as pandemics or other types of emergencies, particularly in their early stages.

### Level of trust in COVID-19 policy

In our study, professionals rated their level of trust in policy as moderate during the first 2 weeks of May 2020, as we had hypothesized. The level of trust was based on professionals' perceptions of the decision-making process, their evaluation of the quality of the guidelines adopted to maintain public health, and the transparency of the decision-making process.

A recent study conducted among Israeli citizens during 2009–2015, years before the COVID-19 outbreak, found similar moderate levels of citizen trust in local government (Mean = 2.76, S.D = 0.37) [24]. Furthermore, a study conducted in Israel and Switzerland during the COVID-19 pandemic, over a similar time frame as the current study, found that Israeli participants reported a lower level of trust than Swiss participants in local government as well as in healthcare institutions, feeling that there was insufficient action taken by these institutions to protect personal health, public health, and wellbeing [22]. This significantly lower trust may reflect the circumstances which preceded the COVID-19 crisis, such as a lack of confidence in the political leadership, which could have decreased trust in the government by the Israeli public. Professionals’ mistrust may have exacerbated public mistrust of health policies, which was particularly low around the world [22], including in Israel [24], even before the COVID-19 outbreak. In contrast, a study focusing on attitudes of nurses during the first wave of COVID-19 found that over 90% of the participants agreed with the policies of the Ministry of Health regarding patient isolation, social distancing, lockdowns, and the obligation to wear masks in public spaces, which attempted to limit the spread of the virus [28]. These high levels of agreement can be attributed to the nature of the work nurses perform in hospitals and the prevailing fear of collapse of the health system due to the high volume of patients at that time, which might explain the nurses’ eager acceptance of the Ministry of Health’s COVID-19 regulations, intended to reduce the pandemic, and prevent the health system from collapsing.

In this context, it is important to note that despite the moderate level of trust, and the significant difference between participants with low and high levels of trust in COVID-19 policy, professionals reported very high personal compliance with the guidelines. A recent report also found similar findings and showed that despite a decline in trust in government and governance in the context of the COVID-19 crisis, there is a high willingness to comply with government guidelines and directives in this regard [29]. Further research is warranted on the contribution of trust, conformism, and other factors to compliance with guidelines among professionals and the public.

### Involvement in decision-making processes

These results suggest that professionals with a higher level of involvement in decision-making processes have a higher level of trust in policy than professionals with a lower level of involvement. Decision making during emergencies requires a non-traditional and flexible approach [30], and an effective response depends on trust and ongoing collaboration between stakeholders [31].

Trust is one of the most important elements for successful collaboration among stakeholders [32]. For example, collaboration during emergencies has been found to raise trust among various governmental levels and between governments [31]. Furthermore, collaboration between researchers and decision makers can lead to improved decision making, policies, practice, and health care outcomes [32]. In these contexts, previous studies have emphasized the positive relationships between public participation in decision-making processes and public trust and confidence both in these processes [33, 34] and in the government [35–37].

The success of public health interventions during the pandemic depends on public trust in experts and on public belief that these experts are involved in the decision making processes for policy development [22]. In order to increase public support on health policy it is critical that the policy is proposed by experts in the relevant field [33, 38]. Therefore, it is critical to involve professionals from relevant fields in the decision-making process [22, 23] during pandemics and other types of emergencies.

The literature emphasizes the critical role of trust in government policy and transparency in decision making in order to obtain an effective crisis response [39, 40] The decision-making process and the internal discourse in the committees should be transparent [41, 42], and based on scientific and epidemiological data [43]. Transparency during the decision-making process allows professionals and other stakeholders who may not be involved in the decision-making process to access the evidence being used to inform management, policy, and decisions [44]. The results of this study indicate that essential aspects of trust in policy include transparency and evaluation of the scientific quality of guidelines adopted to maintain public health. Indeed, a recent study found that during the first wave of COVID-19 Israel managed the pandemic through a centralized and limited team and without satisfactory sharing of information with professionals [42].

The present study found that participants with a low level of involvement in decision making had a lower level of trust in policy compared to those with high level of involvement. Research shows that engaging professionals or their representatives in the policy-making process can increase trust and improve policies and their implementation [22, 23, 32]. Therefore, we recommend and recommended in the past, that the Ministry of Health considers methods to involve professionals in order to improve health policies. However, it should be noted that this study was limited due to its relatively small sample. Furthermore, participants who demonstrated low levels of confidence in policy during the first wave of the pandemic may not have been invited to participate in discussions and decision-making in the first place. Further research is warranted on the contribution of involvement and participation in decision making process on trust in policy.

Indeed, the Ministry of Health and the government made some changes in the decision-making process and professionals' involvement after the first wave of COVID-19. Such measures included: a. More involvement of advisory committees which included governmental and non-governmental professionals, such action was taken to establish the "Magen Israel" (Israel Defender) program, which is a multi-disciplinary plan for management of the COVID-19 health crisis. As part of this program, various mechanisms were established, including the professional Corona Cabinet that involved public health professionals and other experts [45, 46] b. More transparency of advisory committees, which published their protocols, including a public open discussion on COVID-19 vaccination for children, enabling external professional to comment [47] c. Sharing date with professionals and the public, through dashboard and other modes [48]. Their potential impact on trust and on decisions is the subject of a future study.

### Level of trust in COVID-19 policy and its correlations

Our study did not find a correlation between the level of trust in policy and professionals’ socio-demographic factors, such as seniority, age, gender, and level of religiosity. However, there was a difference by profession in trust in policy and the level of trust in policy was correlated with trust in the Ministry of Health, the Prime Minister, and the Minister of Health.

These results suggest that trust in policy is connected to trust in government agencies, and particularly in specific individual officials. A recent paper highlighted that during emergencies, measures adopted to maintain public health require public trust of the information published, which depends on public trust in the authorities as the source of this information [44]. Moreover, trust in the government is a key element required to achieve compliance among citizens regarding measures adopted as public health policies [49]. In addition, a study conducted during the first wave of COVID-19 in Israel found that participants who evaluated the Prime Minister as the most credible spokesperson evaluated the crisis management in a more positive light than did other participants [8]. A recent report found a similar intermediate level of public trust in policy, in the context of evaluating the agencies dealing with the crisis, during the first wave of COVID-19 in Israel; this report also demonstrated a further decline in trust with respect to the second wave [29].

Although there was only initially an intermediate level of trust in policy, Israel’s rollout of COVID-19 vaccinations has been very effective; this gap may reflect high public trust in the safety and efficacy of vaccines and may also reflect high trust in the health system and the government among people over 60 [50]. In addition, despite grave concern about the measures taken to enforce emergency orders, the Israeli Police, whose role is to enforce these orders, has enjoyed a rise in public trust during the COVID-19 pandemic [51]. Our research findings support the current literature and expand the understanding of the relationship between trust in policy and trust in authorities and governments.

### Level of trust in the various agencies dealing with the COVID-19 crisis

Levels of public confidence in politicians and decision-makers were particularly low around the world even before the outbreak of the COVID-19 pandemic [22], and in Israel particularly [24]. With the research showing a very high level of trust in the Association of Public Health Physicians and in hospitals, increased involvement of these leaders (assisting with decision making and vaccine recommendations) could have been used as leverage to increase the level of trust among professionals towards the policy [23]. Moreover, particularly in countries with a diverse population such as Israel, it is extremely important that the health policy information system includes relevant representatives who are highly trusted by the populations they represent, in order to increase the level of trust in policy [43]. Indeed, corrective action was taken during management of the crisis to include public health professionals at a higher level of management, following the establishment of Magen Israel, which included leaders of the Israeli Association of Public Health Physicians and managers of leading hospitals [39].

This study found an association between levels of trust in the various agencies and their leaders and levels of trust in policy. Thus, for example, participants with the highest level of trust in the Prime Minister had higher levels of trust in policy, than those with lower levels of trust in the Prime Minister. Similarly, another Israeli study focusing on public perception of COVID-19 government policy found that participants who regarded the Prime Minister as the most credible spokesman rated the crisis management at a higher level than the other groups [8]. These results are probably related to political personalization that has become a central concept both around the world and in Israel; this is reflected in findings that show that the media’s focus has increased the personal activity of politicians and leaders rather than that of parties and organizations [52, 53].

## Study limitations

The study sample was obtained using purposive sampling. This method does not guarantee a general representation of the target population; however, it was chosen due to the subject of the study and in order to reach the widest possible representation of professionals in the field. Indeed, there was broad participation in terms of public health professionals (physicians, nurses, researchers, lab workers, veterinarians, nutritionists, etc.), education, seniority, and age. The study was small and limited by the response percentage and by the diversity of participants, including those who are not involved in decision-making processes for policy development.

The study was rapidly distributed during the first wave of the COVID-19 pandemic and was conducted according to the COVID-19 restrictions in place at that time. Therefore, it may be assumed that some professionals were engaged in management of the outbreak and therefore did not have the time to answer the survey. To maintain a high level of anonymity and avoid possible identification of the respondents to the questionnaire, the survey did not contain any questions about the actual workplace or affiliation with any organization.

Potential conflicts may be assumed due to the fact that some questions in the survey may have touched on topics within the remit or influence of some of the respondents. Because the entire survey was delivered online and in a completely anonymous manner, no biases related to the interviewees is expected to have occurred.

## Conclusions

The COVID-19 pandemic has placed professionals at the forefront of decision making and highlighted the critical role of their expertise in decision making. This study demonstrates moderate levels of trust among Israeli professionals in national policy and in government agencies during the first wave during March–May 2020, with a higher proportion of public health physicians who had low trust in policy and lower levels of trust among those not involved in decision making. These findings are worrisome, because a low level of trust among professionals may harm cooperation, professional response, and public trust. Actions to increase trust among professionals are essential. Such measures include involving professionals in the decision-making process, increasing the transparency of the process, and basing policy on scientific and epidemiological evidence. Indeed, after the first wave, professionals were greater involved, including participating in, and advising the Corona Cabinet; Increase transparency of advisory committees published their protocols, including a public open discussion on covid-19 vaccination for children as well as sharing data. Further research is needed to examine whether these measurements contributed to increased trust.

The intermediate level of trust in COVID-19 national public health policy among professionals has important implications for future public health emergencies and should be monitored. We recommend conducting periodic surveys of professionals and other groups, to continue to examine the level of trust in policy, particularly during periods of ongoing health crises.

Emergency decision making should be more transparent and inclusive, especially for professionals, in order to increase trust, produce evidence-based policy, and better protect public health.

## Supplementary Information

**Additional file 1.** Study framework.

**Additional file 2.** Survey of trust in COVID-19 policy among public health professionals in Israel.

## Data Availability

The datasets used and/or analyzed during the current study are available from the corresponding author on reasonable request.

## Abbreviation

ISA: Israeli Security Agency
