# Supplementary material for: Trust in COVID-19 policy among public health professionals in Israel during the first wave of the pandemic: a cross-sectional study
Source: Isr J Health Policy Res. 2022 Apr 11;11:20. doi: 10.1186/s13584-022-00529-6 (PMC8995887; doi:10.1186/s13584-022-00529-6)
Supplement: Supplementary file 1 — Additional file 1. Study framework. [file 13584_2022_529_MOESM1_ESM.pptx]

## Slide 1
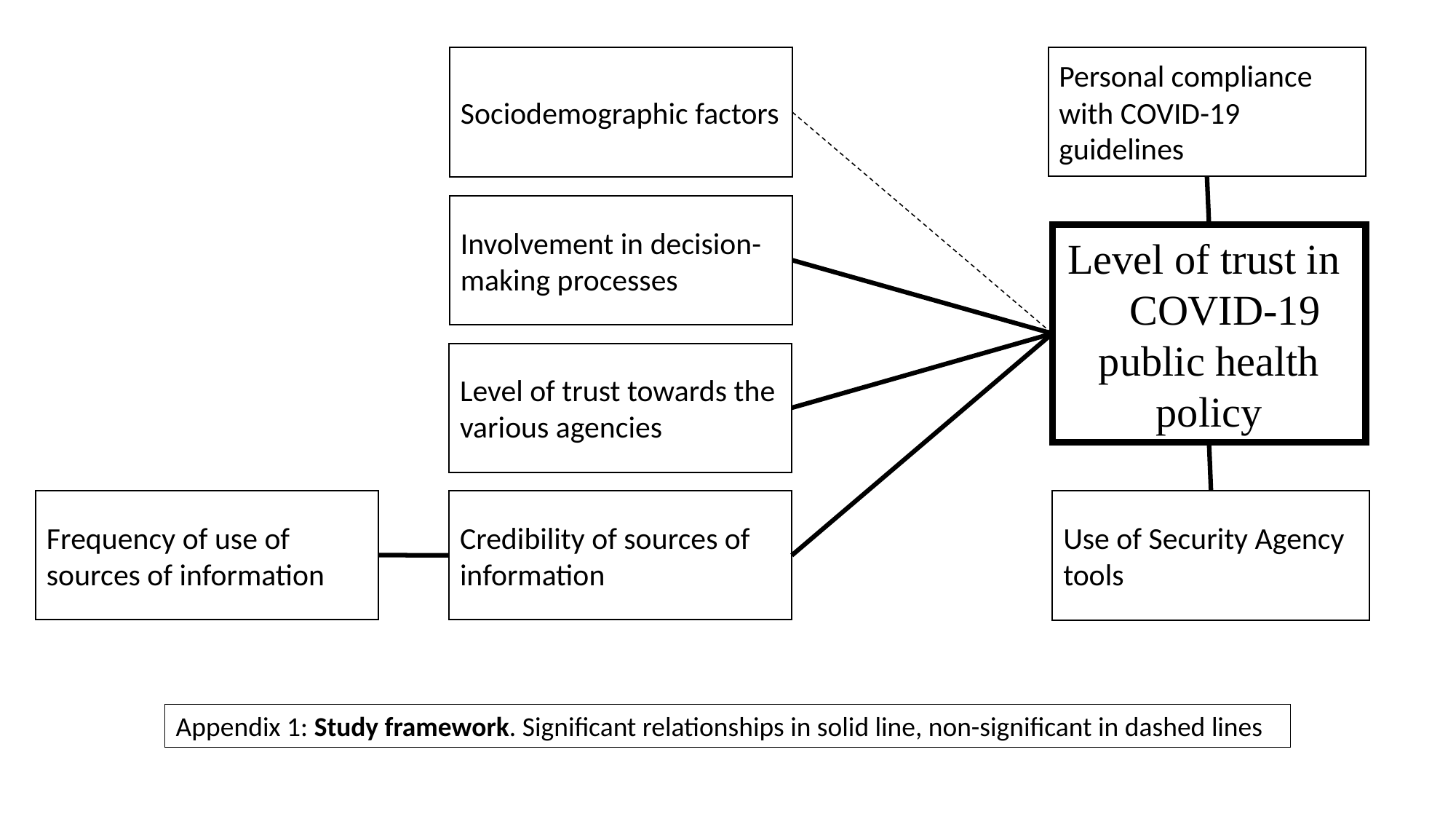

Personal compliance with COVID-19 guidelines
Sociodemographic factors
Involvement in decision-making processes
Level of trust in COVID-19 public health policy
Level of trust towards the various agencies
Frequency of use of sources of information
Credibility of sources of information
Use of Security Agency tools
Appendix 1: Study framework. Significant relationships in solid line, non-significant in dashed lines
