# Supplementary material for: Trust in COVID-19 policy among public health professionals in Israel during the first wave of the pandemic: a cross-sectional study
Source: Isr J Health Policy Res. 2022 Apr 11;11:20. doi: 10.1186/s13584-022-00529-6 (PMC8995887; doi:10.1186/s13584-022-00529-6)
Supplement: Supplementary file 2 — Additional file 2. Survey of trust in COVID-19 policy among public health professionals in Israel. [file 13584_2022_529_MOESM2_ESM.doc]

**Appendix 2**

Survey of trust in COVID-19 policy among public health professionals in Israel

**A.** **To what extent do you trust the reliability of information about the COVID-19 pandemic, from any of the following sources** **(Not at all, to a small extent, to a medium extent, to a high extent, to a very high extent):**

Academic journals

World Health Organization (WHO)

Foreign government websites (e.g. CDC)

Groups online or on social media (e.g. Facebook)

The WhatsApp group "Public Health", run by the Association of Public Health Physicians

Other WhatsApp groups

Israeli media (TV, radio, newspapers, news sites)

Ministry of Health (Israel)

Websites of government ministries in Israel other than the Ministry of Health

**B.** **How often are you updated on information about the COVID-19 pandemic, from any of the following sources (Less than once a week- Every week- Several times a week- Every day- Several times a day)?**

Academic journals

World Health Organization (WHO)

Foreign government websites (e.g. CDC)

Groups online or on social media (e.g. Facebook)

The WhatsApp group "Public Health", run by the Association of Public Health Physicians

Other WhatsApp groups

Israeli media (TV, radio, newspapers, news sites)

Ministry of Health (Israel)

Websites of government ministries in Israel other than the Ministry of Health

**C. This section presents statements addressing your involvement in the decision-making process regarding public health during the COVID-19 pandemic. Please mark the most correct answer for you** **(Not at all, to a small extent, to a medium extent, to a high extent, to a very high extent):**

To what extent is the position of public health experts important in decision-making?

To what extent have public health experts been involved in decision-making?

To what extent have public health experts played the right role in the decision-making processes?

To what extent are you involved in decision-making processes?

Would you like to be more involved in decision-making processes?

To what extent do you participate in the discussions in which decisions are made?

**D. This section presents statements related to public health decisions during the COVID-19 pandemic. Please comment on the following statements (Not at all, to a small extent, to a medium extent, to a high extent, to a very high extent):**

Decisions were made based on data from local and global information

Decisions were made at the right time

The Ministry of Health had an orderly, complete, and clear system of measures to prevent the spread of the virus

The decision-making process was conducted transparently

Decisions give sufficient weight to public health considerations

The outline for ending the quarantine is based on local and global information

The criteria for entering isolation were clear

The measures requested by the Ministry of Health are important for reducing or preventing the spread of the COVID-19.

The plan for returning to school was determined based on local and global information

**E. This section presents statements regarding the degree of personal protection in relation to the instructions given to the public. Please mark the most correct answer for you (Not at all, to a small extent, to a medium extent, to a high extent, to a very high extent**

Did you follow the instruction not to venture more than 100 meters from your home, except for the approved needs?

Did you keep the instruction to wear a mask here as well as in public spaces and at work?

Did you keep a distance of 2 meters when meeting people outside your household?

To what extent have you followed the published guidelines regarding COVID-19?

**F.** **Please describe the level of trust you have in the following bodies’ ability to deal with the COVID-19 pandemic** **(Not at all, to a small extent, to a medium extent, to a high extent, to a very high extent):**

Ministry of Health

Prime Minister

Minister of Health

Government

Knesset

Association of Public Health Physicians

Local authorities

Hospitals

Ministry of Education

Ministry of Finance

Israeli media

**G. This section presents statements related to the decision makers. Please describe your opinion regarding the following statements** **(Not at all, to a small extent, to a medium extent, to a high extent, to a very high extent):**

The Ministry of Health had the ability to take care of the population during the COVID-19 pandemic

Decisions concerning public health were sufficiently transparent at the time of the COVID-19 outbreak

The decision makers in the Ministry of Health acted professionally and responsibly

The decision makers in the Ministry of Health set a personal example in maintaining the guidelines for the public

**H. This section presents statements related to the official guidelines for the public during the COVID-19 pandemic. Please give your opinion regarding the following statements** **(Not at all, to a small extent, to a medium extent, to a high extent, to a very high extent):**

The official guidelines were given clearly and were accompanied by an appropriate information system

The official guidelines were accompanied by information that attested to their level of effectiveness

The official guidelines were based on information and data

The official guidelines were based on professional logic

The official guidelines were given in a way that allowed the public enough time to prepare

**I. This section presents statements related to the use of** **Israeli Security Agency tools for locating patients. Please give your opinion regarding the following statements (Not at all, to a small extent, to a medium extent, to a high extent, to a very high extent):**

There was justification for using Security Agency tools to locate patients in order to reduce / prevent infection by COVID-19

There was a correlation between the measures used and the need to prevent / reduce the spread of COVID-19

There was justification for using Security Agency tools to locate patients even at the cost of violating the rights and privacy of the citizen

In the conflict between the rights of the individual and the need to prevent and reduce the spread of COVID-19, it was necessary to give greater importance to the rights of the individual and the preservation of his/her privacy

Sufficient use was made at the time of the outbreak of the capabilities of the health bureaus

Sufficient use was made at the time of the outbreak of the capabilities of the National Center for Disease Control

**J.1. What is your professional seniority in public health?**

o 1-4 years

o 5-10 years

o 11-15 years

o Over 16 years

**J.2. What is your role?**

a) Public health physician

b) Physician in another field

c) Public health nurse

d) Academic staff

e) Laboratory worker

f) Veterinarian

g) Nutritionist

h) Local authority employee

i) Student

j) ______________________________________________________ Other, please detail

**J.3. What is the highest degree you have received in the field of public health?**

o PhD

o MPH

o MSc

o MHA

o A student in the field of public health

**J.4 Please indicate from which university the degree was awarded?**

The Hebrew University-Hadassah

University of Haifa

Tel Aviv University

Ben Gurion University

Overseas university

**J.5. Gender?**

o male

o Female

**J.6 Year of Birth?**

**J.7 Religion?**

o Jewish

o Muslim

o Christian

o ______________________________________________ Other, please detail

**J.8 Level of religiosity?**

o Secular

o Traditional

o Religious

o Orthodox
